# The role of demethylase AlkB homologs in cancer

**DOI:** 10.3389/fonc.2023.1153463

**Published:** 2023-03-16

**Authors:** Qiao Li, Qingsan Zhu

**Affiliations:** ^1^ Department of Spine Surgery, The First Hospital of Jilin University, Changchun, Jilin, China; ^2^ Department of Orthopedic Surgery, China-Japan Union Hospital of Jilin University, Changchun, Jilin, China

**Keywords:** AlkB homologs, demethylase, cancer, RNA demethylation, DNA demethylation, histone demethylation, therapy

## Abstract

The AlkB family (ALKBH1-8 and FTO), a member of the Fe (II)- and α-ketoglutarate-dependent dioxygenase superfamily, has shown the ability to catalyze the demethylation of a variety of substrates, including DNA, RNA, and histones. Methylation is one of the natural organisms’ most prevalent forms of epigenetic modifications. Methylation and demethylation processes on genetic material regulate gene transcription and expression. A wide variety of enzymes are involved in these processes. The methylation levels of DNA, RNA, and histones are highly conserved. Stable methylation levels at different stages can coordinate the regulation of gene expression, DNA repair, and DNA replication. Dynamic methylation changes are essential for the abilities of cell growth, differentiation, and division. In some malignancies, the methylation of DNA, RNA, and histones is frequently altered. To date, nine AlkB homologs as demethylases have been identified in numerous cancers’ biological processes. In this review, we summarize the latest advances in the research of the structures, enzymatic activities, and substrates of the AlkB homologs and the role of these nine homologs as demethylases in cancer genesis, progression, metastasis, and invasion. We provide some new directions for the AlkB homologs in cancer research. In addition, the AlkB family is expected to be a new target for tumor diagnosis and treatment.

## 1 Introduction

The AlkB family is a member of Fe (II)- and α-ketoglutarate (αKG)- dependent dioxygenase (1–3). Early research indicated that AlkB in E. coli was a DNA repair enzyme (4–6). However, accumulating studies have confirmed that the AlkB homologs are pervasive in eukaryotic cells and act as demethylases on various substrates, including DNA, RNA, and histone (7, 8). There are nine mammalian homologs of the AlkB family, namely ALKBH1-8 and fat mass and obesity-associated protein (FTO) (9–12). Their protein structures all have the function domain of dioxygenase. However, they are present in different locations in the cell, catalyze different substrates, and, therefore, have different biological significance. Some of the nine homologs, such as Alkbh5 and FTO, have been extensively studied as RNA demethylases (13–15). However, others, such as ALKBH4, ALKBH6, and ALKBH7, whose protein structures and function sites are not fully understood.

Methylation and demethylation are ubiquitous epigenetic regulatory mechanisms in cells (16). Epigenetic methylation is the transfer of activated methyl groups to the target chemicals using methyltransferases (writers), leaving the original sequence composition unchanged. Demethylation, on the contrary, is the removal of methyl groups by demethylases (erasers). Those targets are recognized by methylation-dependent binding proteins (readers) (17). The target chemicals include DNA, RNA, and histone, which methylation levels regulated by the “reader, writer, and eraser” are dynamic. In eukaryotic cells, organisms’ development, regeneration, and aging are dynamically regulated by methylation (16). DNA methylation can alter the expression of target genes at the transcriptional level; RNA methylation mainly modulates the processing and decay of RNA; histone methylation can affect the accessibility of genomic DNA. Deregulation of methylation in humans can lead to various diseases, including cancer (15, 18–20). For example, cancer cells undergo epigenetic regulatory changes during proliferation, progression, metastasis, and invasion. The AlkB homologs, as demethylases, are also involved.

This review summarizes the most recent advances in the structural and functional studies of nine AlkB homologs. By reviewing the role of AlkB homologs in tumor-regulating signaling pathways, we reveal that AlkB homologs are a fantastic group of diagnostic and therapeutic targets for tumors. This review aims to translate the cancer-related knowledge of AlkB homologs from the bench side to the bedside.

## 2 A brief history of mammalian AlkB homologs

**Figure 1:**
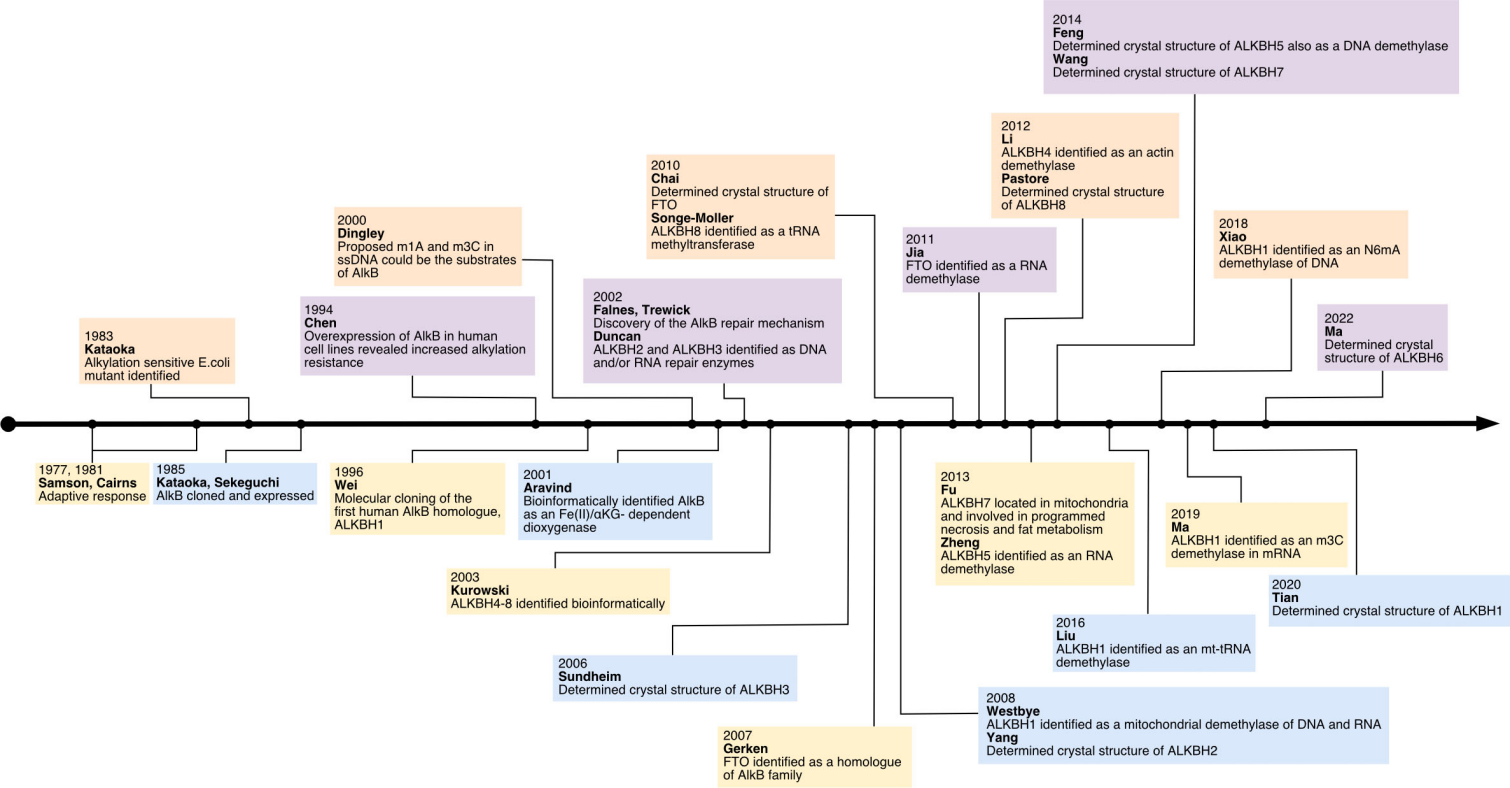
The history of research on human AlkB homologs.

**Figure 2:**
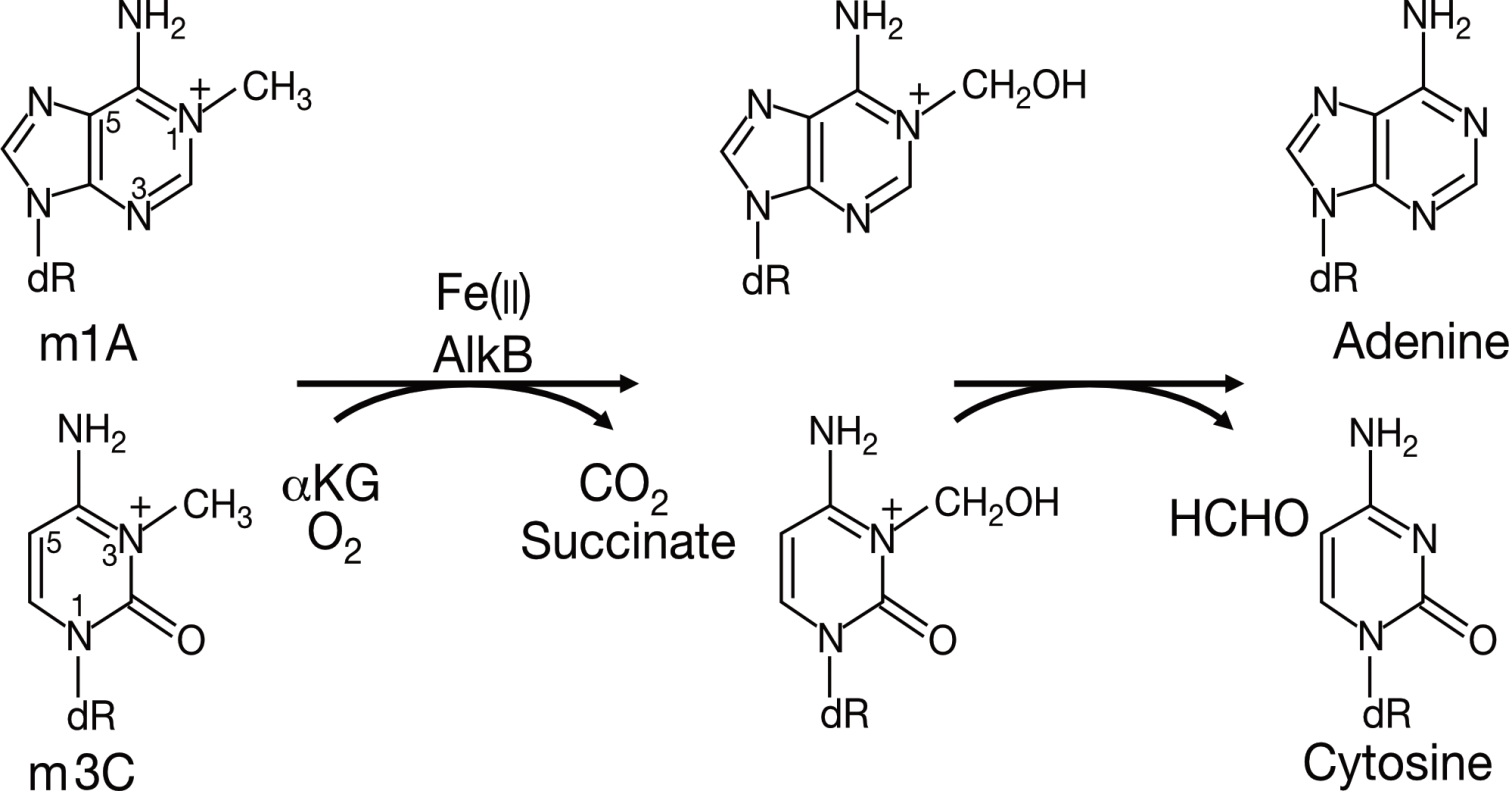
The primary demethylation reaction of AlkB in *E. coli*.

The study of AlkB protein and its human homologs has undergone a long and tortuous process (See **Figure 1**). Initially, the AlkB protein was found to be involved in the process of “the adaptive response” in E. coli to resist the alkylation damage of the DNA (4). In 1977, Samson and Cairns found that after being exposed to a low dose of mutagens such as N-methyl-N’-nitro-nitro-soguanidine (MNNG), the E. coli cells were resistant to the higher levels of mutagens. This resistance was not due to an increase in mutation rate as in the SOS repair system, but rather a decrease in mutation rate (21). This phenomenon was then named “the adaptive response” (22) Subsequently, Kataoka et al. (4) isolated a strain of E. coli lacking the alkb gene that was explicitly sensitive to methyl methane sulfonate (MMS) but not UV light. Two years later, Kataoka (5) used *in vitro* recombination methods to clone and purify the AlkB protein. Then the E. coli AlkB protein was expressed in two human cell lines (23). The AlkB protein from prokaryotes also conferred an alkylation-resistant phenotype in the eukaryotic environment. It suggested that the AlkB had an intrinsic function of alkylation resistance. A dramatic decrease in the reactivation rate of MMS-treated single-stranded DNA phages can be observed in AlkB mutants (24). This result suggests that the AlkB protein acts in the replication forks or at transcriptional regions of the alkylated single-stranded DNA. AlkB was classified as a member of the Fe (II)- and αKG- dependent dioxygenase superfamily by the protein fold-recognition method (1). This bioinformatic conclusion was subsequently validated by two independent researchers that reconstituted AlkB-mediated DNA repair *in vitro*. Simultaneously, these two pieces of research found the mechanism of AlkB repair (2, 3). Specifically, AlkB first uses αKG as a co-substrate and Fe (II) as a cofactor to oxidize the methyl group to produce an unstable hydroxymethyl intermediate, which is then spontaneously released as formaldehyde and unmodified base residues. In this process, αKG is reduced to succinate, and molecular oxygen is the oxidizing agent (25). Initially, N1-methyladenosine (m1A) and N3-methylcytidine (m3C) were considered substrates for AlkB (See **Figure 2**). However, subsequent studies revealed that its substrates are diverse (26). After discovering that AlkB in prokaryotes can also function in eukaryotic cell lines, homologs of AlkB were also identified in mammals. There are nine homologs of the AlkB family, namely ALKBH1-9, and ALKBH9 is commonly named fat mass- and obesity-associated protein (FTO). The first human homolog of AlkB, ALKBH1, was discovered by bioinformatic means by searching an expressed sequence tags (ESTs) database obtained by high-throughput cDNA sequencing (9). The following two human AlkB homologs, ALKBH2 and ALKBH3, were discovered by sequence and folded similarity, functional testing, and complementing the phenotype of the E. coli AlkB mutants (10). Immediately afterward, researchers identified ALKBH4-8 (11). FTO is a protein associated with human body mass index (BMI), adipogenesis, and energy homeostasis (27). Bioinformatic analysis has shown that FTO also belongs to the AlkB family and plays a role as a Fe (II)- and αKG-dependent dioxygenase (12).

**Figure 3:**
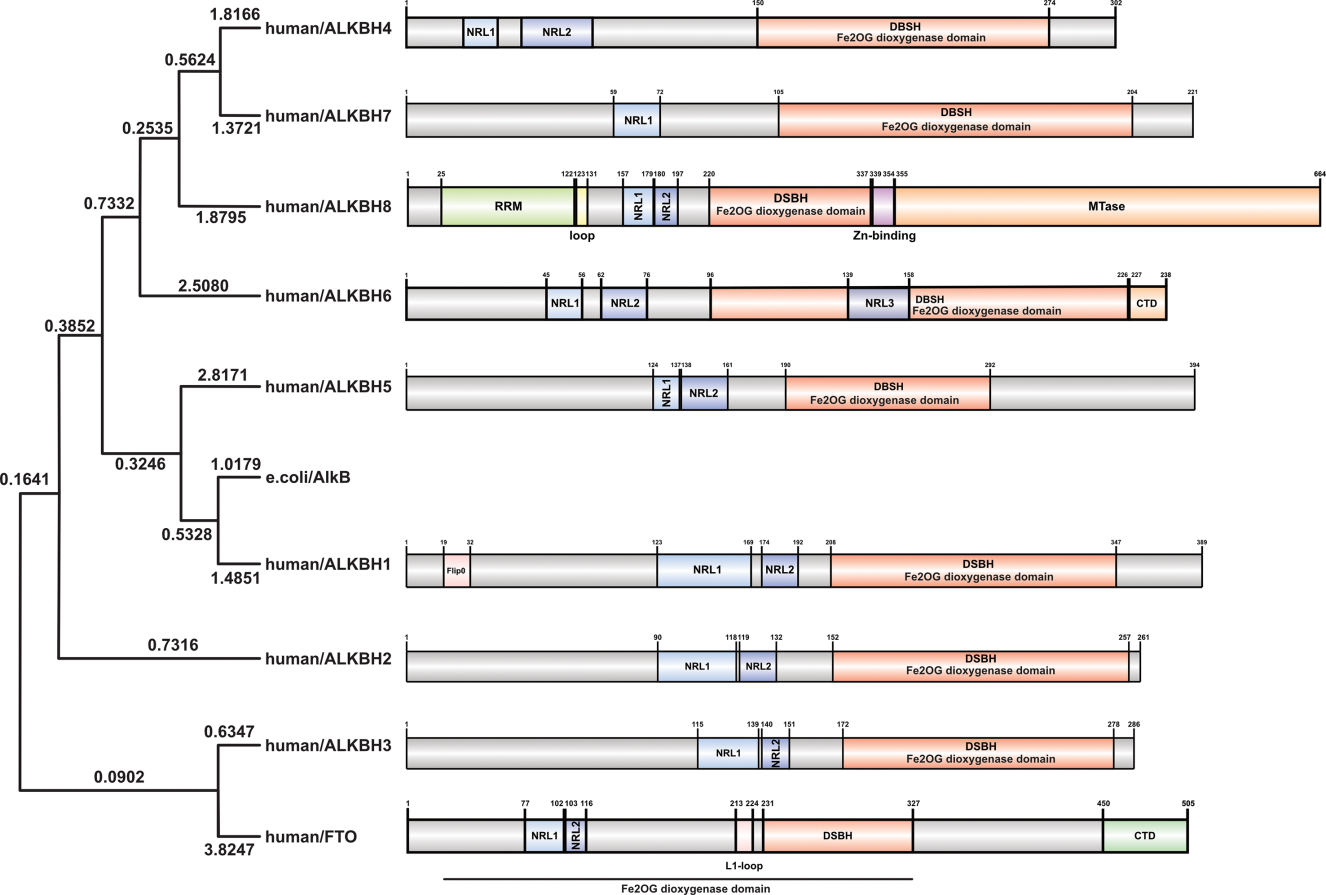
The Phylogenetic tree and function domains of human AlkB homologs. The protein sequences of human AlkB homologs and AlkB of *E. coli* were obtained from the UniProt database (https://beta.uniprot.org) using the first isoform if there were. MEGA11 (28) was used to conduct the analysis. In detail, the sequences were aligned with the ClustalW option. The phylogenetic tree was computed with “Construct/Text Maximum Likelihood Tree,” Bootstrap analysis was performed with 50 replicates. NRL, nucleotide recognition lid; DSBH, double-stranded β-helix domain; RRM, RNA recognition motif; MTase, methyltransferase domain.

Along with gradually revealing their crystal structures, the preferred substrates and physiological functions of AlkB homologs have gotten more attention. Although the nine homologs of the human AlkB protein are structurally conserved compared to bacteria during the course of evolution, the biological functions of each homolog have diverged (See **Figure 3**). Subsequent experiments have indicated that AlkB homologs in mammals have different substrates (DNA, RNA, histone) and locations in the cell (such as the nucleus, cytoplasm, and mitochondria). In addition to its DNA repair function in E. coli, the AlkB homologs in mammals, which functions as demethylases, regulate epigenetic inheritance (7, 8, 29).

## 3 The structures and biological functions of AlkB homologs

The role of AlkB proteins in prokaryotic DNA alkylation damage repair has long been demonstrated; however, the biochemical functions of their nine homologs in eukaryotes, particularly mammals, were elucidated only after their sequence and structure were clarified. Although having different substrates, all nine homologs share a similar catalytic process, that is, utilizing ⍺KG as a co-substrate and Fe(II) as a cofactor to hydroxylate the methyl group on the substrate to produce an unstable intermediate which subsequently releases formaldehyde and unmodified substrate (base or amino acid) (30). However, their crystal structure characteristics determine their ability to bind different substrates and catalyze different reactions (31). Specifically, the lack and variations of the four critical loops in the nine homologs change the shape of the cleft, leading to differences in substrate affinity. At the same time, non-homologous domains are associated with the compatibility of multiple substrates (32) (See **Table 1**). There are five ways for AlkB homologs to bind to substrates: 1) E.coli AlkB and ALKBH1: squeezing double-stranded DNA to turn out its bases to form a single strand to complete the catalytic reaction; 2) ALKBH2: inserting three loops into the grooves of double-stranded DNA to stabilize the double-helical structure of DNA while completing the catalytic reaction; 3) ALKBH3, ALKBH5, and FTO: relatively simple structures with a shallow cleft are well suited for mRNA, but a bit too shallow for double-stranded nucleic acids; 4) ALKBH1, ALKBH8, and FTO: non-homologous structures can specifically bind to RNA; 5) ALKBH4 and ALKBH7: open cleft is more suitable for binding to the relatively irregular spatial structure of proteins. Furthermore, their different intracellular distribution determines their distinct physiological functions.

**Table 1:** The 3D structure of AlkB homologs and their ligands.

Protein	Crystal Structure^a^	Structure Summary^b^	Reference
ALKBH1	PDB ID: 6KSF 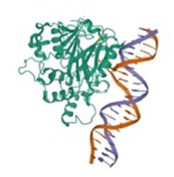	ALKBH1 bound to 21-mer DNA bulge. Ligands: CL, SIN, XL3	(33)
	PDB ID: 6IE2 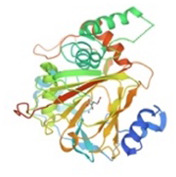	ALKBH1structure Ligands: AKG, MN	(34)
ALKBH2	PDB ID: 3RZH 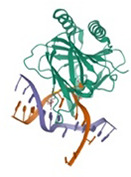	Duplex interrogation by a direct DNA repair protein in the search of damage. Ligands: XL3	(35)
	PDB ID: 3H8X 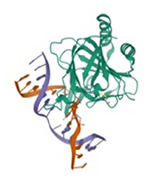	Structure determination of DNA methylation lesions N1mA and N3mC in duplex DNA using a cross-linked host-guest system.	(36)
	PDB ID: 3BUC 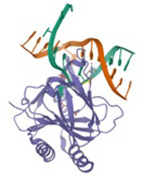	ALKBH2 bound to dsDNA with Mn (II) and 2KG. Ligands: AKG, XL3, MN	(37)
ALKBH3	PDB ID: 6YXQ 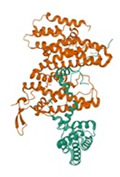	ALKBH3 in a DNA repair complex ASCC3-ASCC2.	(38)
	PDB ID: 2IUW 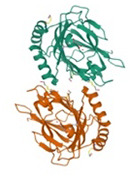	ALKBH3 in complex with FE and AKG. Ligands: AKG, BME, FE	(39)
ALKBH4	AlphaFoldDB: Q9NXW9 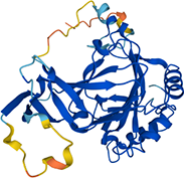	AlphaFold predicted 3D structure.	(40) (41)
ALKBH5	PDB ID: 7WKV 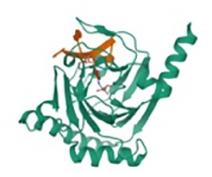	Crystal structure of human ALKBH5 in complex with ⍺KG and m6A ssRNA. Ligands: AKG, MN	(42)
	PDB ID: 4NJ4 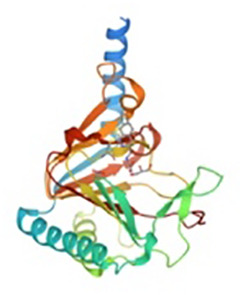	Crystal structure of Human ALKBH5 Ligands: GOL, MN, NO3, UN9	(43)
ALKBH6	PDB ID: 7VJV 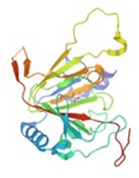	ALKBH6 in complex with AKG and MN Ligands: AKG, MN	(44)
ALKBH7	PDB ID: 4QKD 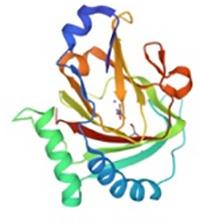	ALKBH7 in complex with AKG and MN Ligands: AKG, MN	(45)
ALKBH8	PDB ID: 3THT 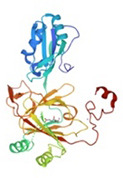	ALKBH8 RRM domain in complex with tRNA as methyltransferase. Ligands: AKG, MN, ZN	(46)
FTO	PDB ID: 3LFM 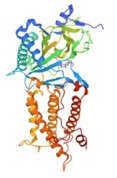	FTO reveals basis for its substrate specificity. Ligands: 3DT, FE2, OGA	(47)
	PDB ID: 5ZMD 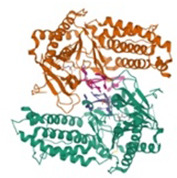	FTO in complex with N6mA ssDNA	(48)

^a^The crystal structures of AlkB homologs are from the PDB and AlphaFold databases. ^b^Ligands: CL, chloride ion; SIN, succinic acid; XL3, propane-1-thiol; AKG, 2-oxoglutaric acid; MN, manganese (II) ion; BME, β-mercaptoethanol; FE, Fe (III) ion; GOL, glycerol; NO3, nitrate ion; UN9, N-[(1-chloro-4-hydroxyisoquinolin-3-yl) carbonyl] glycine; ZN, zinc ion; 3DT, 3-methylthymidine; FE2, Fe (II) ion; OGA, N-oxalylglycine.

### 3.1 ALKBH1

**Figure 4:**
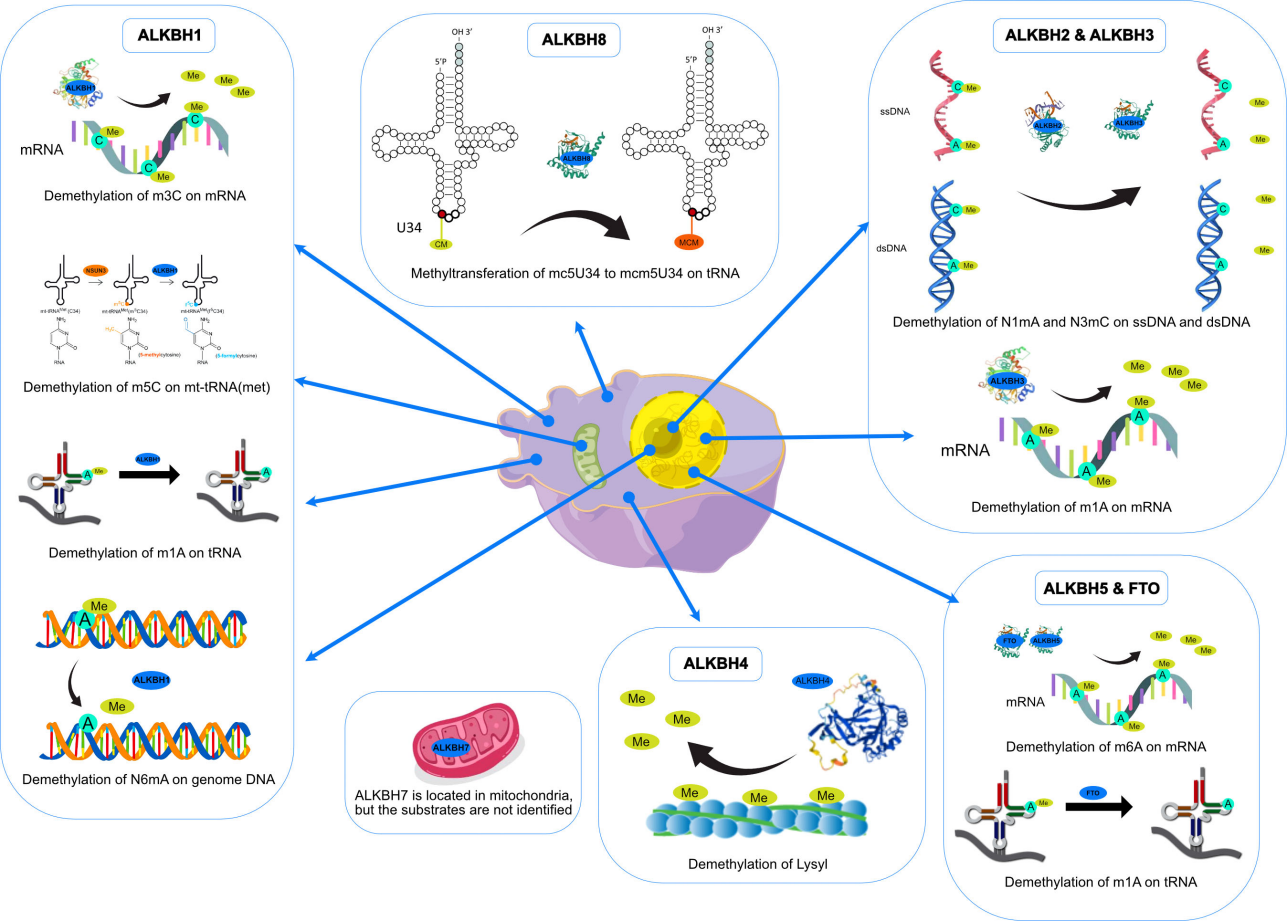
Functions and intracellular localizations of AlkB homologs.

ALKBH1 was the first identified homolog of the human AlkB family by searching the ESTs database obtained by high-throughput cDNA sequencing (9). The human ALKBH1 protein contains 389 amino acids, and its molecular structure (PDB ID: 6IE2, 6IE3) includes a highly conserved double-stranded β-helix (DSBH) fold, which is one of the features of the ⍺KG-dependent dioxygenase superfamily and the core of central catalysis (34). In addition, ALKBH1 contains a unique N-terminal Flip0 and a nucleotide recognition lid (NRL, containing Flip1 and Flip2). Three hydrogen bonds formed by the side chains of Asn220, Asn340, and Tyr222 and three salt bridges formed by Arg338 and Arg344 can further stabilize ⍺KG. The Tyr184 and Glu236 of Flip2 move toward Arg344, creating a stable triangle close to the binding site of ⍺KG through hydrogen bonds. The regular triangle is essential for the demethylation activity of N6mA single-stranded DNA (ssDNA) (see **Figure 3**). Any mutant of the triangles disrupts the demethylation activity but does not reduce the DNA binding affinity. This αKG-induced sizeable conformational change is not observed in other homologs of AlkB (34). The demethylase activity of ALKBH1 mainly shows on nucleic acids, such as DNA, mRNA, and tRNA (49–51). As a demethylase of genomic DNA, the substrate of ALKBH1 is mainly the N6-methyladenine (N6mA) of DNA. CHIP-seq has demonstrated that the N6mA locus is tightly linked to the common heterochromatin marker H3K9me3, thus altering the chromatin accessibility (52, 53). ALKBH1 also demethylates mRNAs containing m3C modifications, thereby affecting protein translation (54). As a tRNA demethylase, ALKBH1 removes N1-methyladenine (m1A) from various tRNAs with a preference for m1A at position 58 (m1A58) on the tRNA stem-loop structure in particular. The tRNAs with m1A modification as the substrates of ALKBH1 can bind preferentially to the polysome to facilitate translation elongation (50). In the mitochondria, the RNA methyltransferase NSUN3 first methylates C34 at the mt-tRNA (Met) wobble position to form m5C34, which ALKBH1 then oxidizes to 5-formylcytosine (f5C34). This mt-tRNA (Met) containing the f5C modification at the wobble position enables the recognition of the AUA codon and the AUG codon, thereby expanding the single mt-tRNA (Met)’s codons recognition encoding methionine during mitochondrial translation initiation and elongation (51). Besides, a study indicated that ALKBH1 also modified the histone H2A K118 and K119 methylation status (55). ALKBH1 is mainly found in the nucleus and mitochondria and is involved in embryonic development, neurodevelopment, and regulation of mitochondrial function (see **Figure 4**).

### 3.2 ALKBH2

ALKBH2 can repair alkylated nucleic acid by direct oxidative demethylation of the methyl groups on the bases of both double-stranded DNA (dsDNA) and ssDNA, with a strong preference for dsDNA (10, 56–60). ALKBH2 contains 261 amino acids, and its crystal structure (PDB ID: 3RZH, 3H8X, 3BUC) shows that the RKK sequence (Arg241-Lys243) and residues Arg198, Gly204, and Lys205 take charge of DNA binding (see **Table 1**). In addition, the recognition of N1mA by ALKBH2 is accomplished by residues in five active sites. Phe124 and His171 can stack against N1mA, while Tyr122, Glu175, and Asp174 form an extensive hydrogen bonding network with a water molecule (35, 36) (see **Figure 3**). ALKBH2 has a DSBH structure common to the AlkB family that flips the methylated bases into a deep active site pocket and subsequently uses Fe (II) and 2-oxoglutarate as cofactors to remove the methyl group and regenerate the unmethylated bases (37). Proliferating cell nuclear antigen (PCNA) is eukaryotic DNA polymerases’ primary DNA sliding clamp. It is involved in DNA damage signaling and repair, cell cycle progression, and cellular stress responses (61). The specific structure of ALKBH2 can bind to PCNA to repair methylated bases during the S phase of cell mitosis (62). ALKBH2 can repair both monoalkylated adducts, such as N1mA, N3mC, and N3-ethylthymidine (N3EtT), and higher-order alkylated adducts, such as exocyclic bridge adducts known as etheno- adducts, including 1, N6-ethenoadenine (ϵA), 3, N4-ethenocytosine (ϵC), and 1, N2-ethenoguanine (1, N2-ϵG) of the DNA (10, 56–60, 62, 63). Damage to ribosomal DNA (rDNA) will severely affect DNA replication and transcription, leading to the generation of single-strand and double-strand breaks. ALKBH2 promotes rDNA transcription by repairing the alkylation damage caused to the rDNA gene under alkylation stress (60). ALKBH2 is only found in the nucleus (10, 56, 61), specifically in the nucleolus and nucleoplasm (60) (see **Figure 4**).

### 3.3 ALKBH3

ALKBH3 was identified as a homolog of the AlkB family along with ALKBH2 and can remove m1A and m3C from methylated polynucleotides (10). ALKBH3 prefers ssDNA and RNA compared to ALKBH2, which picks dsDNA (39). ALKBH3 protein contains 286 amino acids. It also has the DSBH and NRL structural domains characteristic of the AlkB family (PDB ID: 6YXQ, 2IUW) (See **Table 1**). The β4 and β5 strands in the ALKBH3 protein form a hairpin structure that creates a lid over the active site. A positively charged groove traverses the active area between the β4 and β5 hairpins and the jelly roll core, forming a DNA/RNA binding cleft. Three residues, His191, Asp193, and His257, form an iron binding site with the co-substrate αKG. Tyr143 forms a hydrogen bond with N6mA, and Leu177 acts as the core of the demethylation reaction, while Arg131 interacts with N3mC (39) (See **Figure 3**). ALKBH3 strongly prefers repairing alkylated ssDNA containing N1mA and N3mC by oxidative demethylation (10, 56–58, 64). However, ALKBH3 can also promote unwinding dsDNA to generate ssDNA by interacting with the activating signal integration complex (ASCC), which processes this region’s N3mC (65). ASCC3 is the largest subunit of ASCC and a critical node in the alkylation-specific damage response. It can sense ubiquitin-dependent signals and recruit ALKBH3 (66). ALKBH3 can also repair ϵC adducts in ssDNA (59). As an RNA demethylase, ALKBH3 demethylates m1A, an internal epigenetic modification of mRNA that is highly enriched in the 5’-untranslated regions (UTRs) and near the start codon (67, 68). ALKBH3 can also catalyze 5-methylcytosine (5mC) *in vitro*, a crucial epigenetic biomarker in DNA CpG islands, affecting gene expression (69). In addition, ALKBH3 has a direct protein-protein interaction with human RAD51C, and such interaction stimulates the ALKBH3-mediated repair of methylated bases located within the 3’- tail DNA, which is a substrate for RAD51 recombinase (70) (See **Figure 4**).

### 3.4 ALKBH4

Since identifying ALKBH4 as a member of the AlkB family, its structure and physiological functions have yet to be thoroughly studied (11). Human ALKBH4 has 302 amino acids; however, its crystal structure has not been resolved (see **Table 1**). The structure predicted by AlphaFold (40, 41) (AlphaFoldDB: Q9NXW9) suggests that ALKBH4 also has a DSBH structure that is shared by the Fe(II)/⍺KG-dependent dioxygenase superfamily, and His169, Asp171, and His154 in this structure can bind Fe(II), whereas Arg265 is responsible for binding αKG (see **Figure 3**). ALKBH4 has been predicted to have protein-binding ability. Evidence shows that ALKBH4 can bind to actin and remove the methyl group at Lys84 (K84me1). The demethylated state of actin K84me1 is necessary to maintain the actomyosin dynamics, which support the proper entry of the cleavage furrow during cell division and migration. ALKBH4 is a vital regulator of actomyosin processes (71). In zebrafish, ALKBH4 is associated and co-exists with ATRN to influence the embryonic developmental epiboly by regulating actin formation (72). In another study, the knockdown of ALKBH4 in mice did not affect the entry of germ cells into meiosis, but spermatogenesis was arrested at the pachytene stage. ALKBH4 was located in the nucleus of spermatogonia and Sertoli cell nucleosomes. Homologous chromosome pairing, synaptonemal complex formation, and long-distance chromosome movement depend on actin and myosin, and ALKBH4 affects cell meiosis by regulating actomyosin dynamics in mammalian meiosis (73). ALKBH4 in the nucleolar structures of Sertoli cells, spermatogonia, and primary spermatocytes is involved in essential physiological processes, including cytokinesis and cell motility. Lack of ALKBH4 in early embryo preimplantation is fatal (see **Figure 4**).

### 3.5 ALKBH5

ALKBH5 has been extensively studied as a mammalian mRNA demethylase (74). ALKBH5 contains 394 amino acids, and its crystal structure also includes the conserved DSBH core fold and NRL. (PDB ID: 7WKV, 4NJ4) (see **Table 1**). The conserved ALKBH5 residue Arg130 in NRL1 and the two basic residues of NRL2, Lys147 and Arg148, are essential for substrate recognition through interaction with the phosphate backbone of RNA substrates. Besides, Arg30, Tyr139, His204, and αKG form a positively charged pocket where N6-methyladenine (m6A) can be positioned. This structure allows ALKBH5 to catalyze the demethylation of m6A both on ssRNA and ssDNA (43, 75, 76). (see **Figure 3**). In higher eukaryotes, demethylation of m6A is mRNA’s most prevalent epigenetic modification. In ALKBH5-deficient male mice, the m6A level is elevated, leading to the apoptosis of meiotic metaphase-stage spermatocytes and thus impairing fertility (74). In the testes of ALKBH5-deficient mice, there are 1551 genes with varying degrees of differential expression, which cover a wide range of functional categories. This suggests that reversible mRNA m6A modifications in mammalian cells are involved in many physiological functions. ALKBH5 can also demethylate N6mA in ssDNA *in vitro* (75). There is evidence that ALKBH5 is a hypoxia-induced gene acting through hypoxia-inducible factor 1α (HIF-1α), which is unique among members of the AlkB family (77). ALKBH5 is found only in nuclear speckles (74, 77) (see **Figure 4**).

### 3.6 ALKBH6

Among all the AlkB Homologs, ALKBH6 is the least informed. A recent study has resolved the crystal structure of ALKBH6 (PDB ID: 7VJV) and analyzed its potential biological functions (44) (see **Table 1**). Human ALKBH6 contains 238 amino acids and, as a member of the AlkB family, also includes the NRL domain for substrate recognition and the conserved DSBH domain, in which His114, Asp116, and His182 stably coordinate the metal ion. The NRL domain is the critical structure for AlkB family proteins to bind and immobilize substrates. It is a less conserved structure among AlkB homologs due to differences in the substrate’s selectivity. The NRL1 and NRL2 constitutive NRL structural domains of ALKBH6 are distinctly different from the NRL structural domains of other human AlkB homologs. In addition, the NRL3 domain present in the DSBH structural domain may have a variety of unreported functions, such as distinguishing between double-stranded nucleic acids, binding to other proteins, and blocking active centers (see **Figure 3**). Structural analysis and substrate screening suggested that ALKBH6 may also function as a nucleic acid demethylase by revealing how it distinguishes between different types of nucleic acids. ALKBH6 is mainly distributed in the cytoplasm and nucleus, with the highest expression in the testis and pancreas (11, 78). A study showed that a deficiency of ALKBH6 in human pancreatic cancer (PC) cells increased alkylation-induced DNA damage and thus significantly reduced cell survival. In contrast, bioinformatics analysis of the The Cancer Genome Atlas (TCGA) database showed that overexpression of ALKBH6 led to better survival outcomes in pancreatic cancer patients. It suggests that ALKBH6 may be essential in maintaining genomic integrity, especially in cancer (79) (see **Figure 4**).

### 3.7 ALKBH7

ALKBH7 is the only member of the AlkB family that is found exclusively in mitochondria. Human ALKBH7 (PDB ID: 4QKD) contains 221 amino acids, and its catalytic core contains a conserved DSBH fold domain, which is characteristic of the Fe (II)/αKG-dependent dioxygenase superfamily (see **Table 1**). However, the most crucial structural distinction of ALKBH7 lies in the motif of the NRL. In contrast to the structures of other AlkB homologs, ALKBH7 lacks NRL2. This defective NRL results in a solvent-exposed active site and lack of nucleobase binding capacity. Moreover, Asp182, Glu183, Glu184, and Glu189 bind to Glu150, Glu153, Glu156, and Glu178 of other loops to form the ALKBH7’s negatively charged surface, and the cleft into the active site with Glu62, Asp64, Asp67, Glu75, and Glu77 were also negatively charged. The negatively charged surface of ALKBH7 suggests that it prefers to bind to positively charged molecules, such as lysine/arginine-rich proteins (45) (see **Figure 3**). ALKBH7 was initially reported to localize both in the cytoplasm and nucleus (78). However, it was later shown to localize in the mitochondrion (80). Unlike other human AlkB homologs that have been shown to exert protective effects upon DNA exposure to alkylating agents, ALKBH7 promotes cell death upon exposure to alkylating or oxidizing agents by inhibiting mitochondrial function and reducing the regeneration of critical bioenergetic metabolites such as NAD+ and ATP (80). This suggests that ALKBH7 may be involved in cell necrosis or apoptosis. The modulation of necrotic cell death by ALKBH7 after alkylating agent exposure proved specific for both cell type and sex. ALKBH7 acts in some tissues, such as the retina and cerebellum, but does not affect others. This may be due to the different proportions of various cell death modes in each tissue type. In addition, the cerebellar response to alkylation-induced injury showed gender dimorphism. Compared to wild-type male mice, ALKBH7-deficient males showed more protection against MMS-induced necrotic damage to cerebellar tissue, whereas ALKBH7-deficient females provided no protection and were even sensitive to the injury (81). Moreover, ALKBH7 knockout mice exhibited an abnormally high level of body fat, suggesting a role for ALKBH7 in fat metabolism and obesity (82). A proteomic analysis study revealed that ALKBH7 is engaged in protein stabilization and cell immunity, as well as cell proliferation, lipid metabolism, and programmed necrosis by regulating the expression of HMGN1, HMGN2, PTMA, PTMS, and UQCRH (83). A recent study identified ALKBH7 as an RNA demethylase that regulates nascent mitochondrial RNA processing and mitochondrial activity (84). Specifically, ALKBH7 demethylates Ile m2^2^G and Leu1 m1A within the pre-tRNA region in nascent polyclonal mitochondrial RNAs, thereby regulating their processing and structural dynamics (See **Figure 4**).

### 3.8 ALKBH8

Human ALKBH8 (PDB ID: 3THT) consists of 664 amino acids. In addition to the conserved NRL domain and DBSH domain of Fe (II)/⍺KG dioxygenase, it also has an RNA recognition motif (RRM) domain and a methyltransferase (MTase) domain at the N- and C- terminal, respectively (see **Figure 3** and **Table 1**). Although ALKBH8 has both Fe(II)/⍺KG and MTase, two functionally opposite structural domains, both of its catalytic substrates were shown to be super-covalent modifications of wobbly nucleotides in specific tRNA anticodon loops (85–88). The MTase domain of ALKBH8 acts in combination with the accessory protein Trim112 to methylate 5-carboxymethyluridine (cm5U) in the tRNA wobble position to produce 5-methoxycarbonyl methyluridine (mcm5U). This modification is the final and most critical post-transcriptional modification of the specific tRNA anticodon loop (85, 88, 89). ALKBH8 has a preference when binding tRNA, such as favoring tRNA (Arg), tRNA (Gly), and tRNA (Glu) but not tRNA (Lys) (86, 87). In addition, there is a unique structural Zn(II) binding site between the C-terminus of the DSBH domain and the MTase domain, and this site binds Zn(II) to stabilize the DSBH domain binding Fe(II) and ⍺KG (46, 86, 87). Although ALKBH8 knockout mice do not exhibit any abnormalities, the selenocysteine-specific tRNA (tRNASec) is aberrantly modified due to the lack of mcm5U (85). The regulation of the selenocysteine protein by ALKBH8 protects cells against the oxidative stress response (90). Several studies have now established a link between ALKBH8 deficiency and several diseases. The homozygous truncated ALKBH8 mutation causes wobbly uridine modification disorder and mental retardation (91). ALKBH8 is involved in aging, regulating stress response genes, and reprogramming mitochondria by regulating selenoprotein levels. However, ALKBH8-deficient mouse embryonic fibroblasts (MEFs) die due to glycolytic inhibition (92). ALKBH8 may also inhibit apoptosis and promote cell survival and angiogenesis (93) (See **Figure 4**).

### 3.9 FTO

FTO has currently been identified to catalyze the demethylation of multiple substrates, such as m6A and cap m6Am in mRNA, m6A and m6Am in snRNA, and m1A in tRNA (94–98).

The human FTO protein (PDB ID: 3LFM, 5ZMD) contains 505 amino acids. Its crystal structure contains an N-terminal domain (NTD, residues 32-326) whose catalytic core is the conserved DSBH structure of the AlkB family and a C-terminal structural domain (CTD, residues 327-498) that has a crucial role in stabilizing the conformation of the NTD (see **Figure 3** and **Table 1**) (47, 99). In the NTD core catalytic structure, residues His231, Asp233, and His307 chelate with Fe(II), and Arg316 and Arg322 form salt bonds with NOG (12, 100). Two highly conserved residues, Tyr108 and His231, can sandwich the nucleobase loop of 3mT between the side chains, while Leu109 and Val228 wrap around the sugar loop of 3mT to form a hydrophobic interaction. These three hydrogen bonds facilitate the interaction of 3mT with FTO (47). There are many epigenetic modifications of mRNA, but the demethylation of m6A is the most widespread and vital (94, 96–98, 101). Demethylation of m6A by FTO affects the expression and stability of mRNA (96). In mRNA and U6 snRNA, FTO prefers to bind cap m6Am rather than m6A in sequence, and this activity preference arises from differences in the sequence and structure of RNA rather than m6Am and m6A ribose loops (48, 95, 96).

The mRNA 5’-cap is essential for mRNA stability, while FTO increases the mRNA’s susceptibility to decapping by demethylating m6Am in the 5’-cap, which affects mRNA stability (95). The demethylation of m1A in various tRNAs is another function of FTO in protein synthesis (96). As an RNA demethylase, FTO is involved in many biological procedures, such as stem cell fate determination, adipogenesis, stress response, energy homeostasis, and carcinogenesis (94–98, 101). Depletion of FTO induces chromosomal instability and G2/M arrest in mouse spermatogonia. Mechanically, FTO targets Mad1, Mad2, Bub1b, Cdk1, and Ccnb2 to regulate the expression of mitotic checkpoint complex and G2/M regulators (102).

As an adipogenesis-associated regulatory protein, FTO acts through cell cycle regulation. Knockdown of FTO can elevate cyclin A2 (CCNA2) and cyclin-dependent kinase 2 (CDK2) mRNA m6A levels, thereby inhibiting their protein synthesis. Both proteins interact with YTHDF2 to participate in cell cycle progression, while the downregulated proteins prolong the cell cycle, inhibiting fat synthesis (103). Another experiment showed that knockdown of FTO resulted in mRNA instability of signal transducer and activator of transcription (STAT1) and peroxisome proliferator-activated receptor-γ (PPAR-γ), reducing protein translation and inhibiting NF-κB signaling pathway, which in turn impeded macrophage activation (104). Overexposure to glucocorticoids can induce nonalcoholic fatty liver disease (NAFLD), which is mediated by the glucocorticoid receptor (GR). GR-dependent FTO transactivation and m6A demethylation on lipogenic gene mRNA can activate lipogenic genes and lipid accumulation in hepatocytes. Knockdown of FTO effectively attenuated dexamethasone-induced fatty liver in mice (105). Splicing factor proline/glutamine-rich (SFPQ) is a protein that plays a role in pre-mRNA splicing, RNA processing, and regulation of gene expression. A recent study has shown that the significant RNA binding proteins SFPQ and FTO are close to the transcriptome and that SFPQ can promote m6A demethylation by recruiting FTO to specific RNA sites (106). FTO can also oxidatively demethylate 3mT in ssDNA and 3mU in ssRNA *in vitro*, with the latter being slightly more efficient than the former, while the activity against 3mT in dsDNA is negligible (107). FTO shuttles between the nucleus and cytoplasm *via* its specific N terminus, which is compatible with its function of regulating mRNA m6A methylation levels (108) (See **Figure 4**).

## 4 The roles of AlkB homologs in cancer

**Figure 5:**
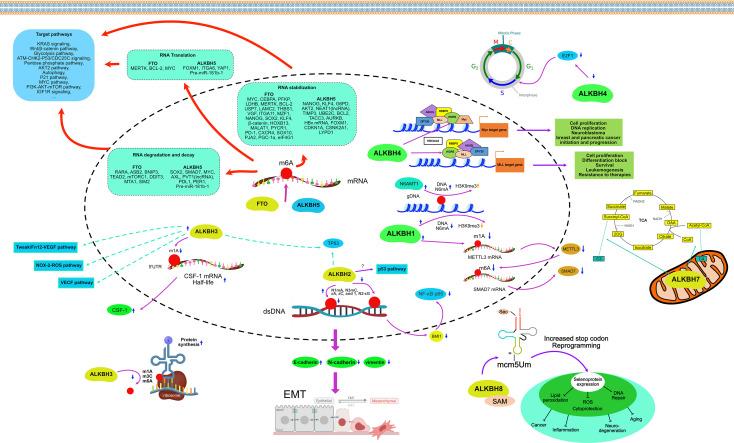
Mechanism of AlkB homologs in the regulation of cancer.

AlkB protein, first discovered in E. coli, can resist alkylation damage and repair DNA. However, with the discovery of its homologs in eukaryotes, especially in humans, its role as a demethylase became increasingly prominent. Methylation and demethylation as the most common and primary pair of epigenetic modifications that regulate tumorigenesis, tumor development, migratory and invasive properties of tumor cells, and resistance to antitumor drugs have received attention incrementally (16, 109–112). As the catalytic substrates of AlkB homologs continue to expand, their research has also accumulated rapidly in the last decades, including single nucleotide polymorphisms, signaling pathways, and bioinformatic analyses (14, 32, 113–115). The epigenetic regulatory roles of AlkB homologs in cancers are summarized separately below (See **Figure 5**).

### 4.1 ALKBH1

Among the multiple substrates of ALKBH1, N6mA of genomic DNA has the most critical effect on cancer. In glioblastoma, levels of N6mA were significantly elevated, and N6mA co-localized with heterochromatin histone modifications (mainly H3K9me3). Down-regulation of ALKBH1 led to an increased N6mA level in genomic DNA, which coordinated with H3K9me3, reduced chromatin accessibility, and silenced some oncogenes’ transcription. Targeted knockdown of ALKBH1 in a patient-derived human glioblastoma model inhibited the proliferation of tumor cells, which in turn prolonged the survival of mice (52). However, in a study of tongue squamous cell carcinoma (TSCC), increased levels of genomic N6mA were found in the TSCC tissues and cultured cells. Knockdown of ALKBH1 resulted in increased levels of N6mA in genomic DNA, which conversely enhanced tumor colony formation and cell migration.

In contrast, the knockdown of N6AMT1 and METTL4, methyltransferases commonly considered to act opposite to ALKBH1, decreases genomic N6mA leading to inhibition of TSCC cell colony formation and cell migration capacity (116). A similar pattern can be seen in a study of hepatocellular carcinoma (HCC). Overexpression of N6AMT1, a methyltransferase, increased levels of N6mA, resulting in increased HCC cell viability, decreased apoptosis, and enhanced ability to migrate and invade, whereas overexpression of ALKBH1 induced the opposite effect (117). Moreover, overexpression of ALKBH1 reversed the inhibitory effect of miRNA-339-5p, an upstream gene of ALKBH1, on the proliferation and migration ability of gastric cancer (GC) (118). In addition to the role of a genomic DNA N6mA demethylase, ALKBH1 can bind mRNA and act as a demethylase. ALKBH1 is upregulated in lung cancer tissues and cells, and silencing of ALKBH1 significantly inhibits the invasion and migration ability of lung cancer cells *in vitro*. It is greatly enhanced by the overexpression of ALKBH1 (119).

Similarly, overexpression of ALKBH1 has been proven to promote metastasis in both *in vivo* and *in vitro* experiments in colorectal cancer (CRC) (120). The mechanism of this effect is that ALKBH1 can modify m1A in the mRNA of the methyltransferase 3, N6-adenosine-methyltransferase complex catalytic subunit (METTL3). Knockdown of ALKBH1 increased METTL3 mRNA m1A levels resulting in reduced protein translation. Reduced METTL3 expression was further followed by increased m6A demethylation of SMAD7 mRNA, resulting in enhanced tumor migration and invasion. Cell migration and invasion defects caused by the depletion of ALKBH1 and METTLE3 can be significantly reversed by silencing SMAD7 (120).

### 4.2 ALKBH2

ALKBH2, a dioxygenase that repairs alkylated nucleic acid bases, mainly exists in the nucleolus and nucleoplasm. Both dsDNA and ssDNA can be the substrates of ALKBH2, but it prefers dsDNA. ALKBH2 can repair N1mA, N3mC, and ϵA, ϵC, and 1, N2-ϵG with etheno adducts (10, 56–60, 62). ALKBH2 expression was downregulated in primary GC and GC cell lines. Overexpressing ALKBH2 could induce cell cycle G (1) arrest and thus significantly inhibit the proliferation of GC cells. Conversely, the knockdown of ALKBH2 could promote cell cycle progression as well as the growth of cancer cells (121). However, in some cancers, the regulation of ALKBH2 is reversed. In the human urothelial carcinoma cell line, the induction of G (1) cell cycle arrest was regulated by ALKBH2 through MUC1, a downstream transmembrane mucin protein. Downregulation of ALKBH2 inhibits epithelial-to-mesenchymal transition (EMT) by increasing E-cadherin and decreasing vimentin expression (122). The same regulatory effect was shown in CRC cell lines, where knockdown of ALKBH2 inhibited CRC cells’ proliferation and invasive capacity by increasing E-cadherin and decreasing N-cadherin expression. Silencing ALKBH2 inhibited BMI1 expression, which reduced nuclear accumulation of NF-κB p65 protein and luciferase activity of NF-κB p65 (123). ALKBH2 expression levels are elevated in human glioblastoma multiforme (GBM) and established GBM cell lines. GBM cell lines exposed to the methylating drug temozolomide upregulate ALKBH2 expression levels, and overexpression of ALKBH2 inversely increases cancer cell resistance to temozolomide and methanesulfonate. Knockdown of ALKBH2 then increased the sensitivity of cancer cells to both of these drugs. p53 pathway may be involved in this regulation (124). TP53 in glioma cells increased ALKBH2 expression by binding to the ALKBH2 promoter (125).

### 4.3 ALKBH3

ALKBH3 and ALKBH2 are located in the same clade of the phylogenetic tree and have similar enzymatic functions of DNA (7). Unlike ALKBH2, ALKBH3 is more inclined to bind N1mA and N3mC of ssDNA and m1A of mRNA highly enriched in UTRs and near the start codon (10, 56–58, 64, 67, 68). ALKBH3 and prostate cancer antigen-1 (PCA-1) are identical and abundantly expressed in many human cancers (126, 127). Due to the possibility that MAQ probes can measure ALKBH3 activity to measure patient response to treatment, many studies have linked ALKBH3 to tumor growth (128). In the hormone-dependent prostate cancer cell line DU145, silencing ALKBH3 significantly induced apoptosis, inhibiting its tumorigenicity, tumor-forming size, and anchor growth *in vivo* (129). On this basis, inhibitors of ALKBH3 were synthesized and proved to inhibit the growth of DU145 *in vivo* (130, 131). The knockdown of ALKBH3 in human lung adenocarcinoma cells induces p21 (WAF1/Cip1) and p27 (Kip1) expression, leading to cell cycle arrest, senescence, and potent inhibition of cell growth *in vitro*. Intraperitoneal injection of ALKBH3 siRNA and atelocollagen in nude mice inhibited the growth and spread of peritoneal tumors (127). In non-small cell lung cancer (NSCLC) cell line A549, TP53 gene status was correlated with the phenotype of A549 cells after ALKBH3 knockdown. TP53 knockdown shifts A549 cells from cell cycle arrest to apoptosis after ALKBH3 knockdown (132). The cytokine CSF-1 can contribute to poor prognosis in ovarian and breast cancers. ALKBH3 acts as an mRNA m1A demethylase that regulates the stability of CSF-1 mRNA. Specifically, ALKBH3 increases the half-life of CSF-1 mRNA by demethylation of m1A on the 5’UTR, which does not affect the translation efficiency of mRNA because the 5’UTR maps near the translation initiation site (133). However, in another study, the inactivation of ALKBH3 due to CpG promoter methylation led to reduced survival time in breast cancer patients (134). The expression of ALKBH3 was upregulated to varying degrees in PC, HCC, renal cell carcinoma (RCC), and urothelial carcinoma (135–138). The upregulation degree of ALKBH3 was correlated with tumor proliferation ability, prognosis, and survival time. Knockdown of ALKBH3 inhibits tumor anchorage growth and migration. The signaling pathways such as VEGF (pancreatic cancer) (135), NOX-2-ROS, and Tweak/Fn12-VEGF (urothelial carcinoma) (137) were involved in the ALKBH3 regulation.

In addition, tRNAs with m1A, m3C, and m6A demethylated by ALKBH3 in cancer cells increase the efficiency of protein synthesis (139). The tRNAs with m1A and m3C demethylated by ALKBH3 can produce tRNA-derived small RNAs (tDRs), thereby promoting cancer development. Conservative tDRs can be used as biomarkers for cancer diagnosis (140, 141).

### 4.4 ALKBH4

The crystal structure and physiological function of ALKBH4 have yet to be thoroughly investigated. The only evidence suggests that ALKBH4 is a demethylase of actomyosin K84me1. Regulation of actomyosin by ALKBH4 can affect cell mitosis, meiosis, and cell motility. A study on NSCLC showed that ALKBH4 is highly expressed in NSCLC cells. Knockdown of ALKBH4 downregulated the expression of E2F1, a vital regulator of the G1/S phase transition during cytokinesis in NSCLC cells (142). However, in another study on CRC, ALKBH4 acted as an EMT suppressor gene and was significantly downregulated in CRC patients.

Further studies demonstrated that ALKBH4 could bind competitively to WDR5, a critical constituent of the histone methyltransferase complex, which reduced the histone methylation levels H3K4me3 on target genes, including miR21 (143). It suggests that ALKBH4 affects the development of cancer cells in different cancers through different pathways. Bioinformatics analysis indicated that high expression levels of ALKBH4 were negatively correlated with overall survival and disease-free survival in patients with HCC (144). Increased expression of ALKBH4 was also positively correlated with the pathological stage of HCC (144).

### 4.5 ALKBH5

ALKBH5 was found to have the catalytic function of removing m6A on single-strain RNAs (74). The methylation status of mRNA, the most common ssRNA in cells, can affect pre-mRNA processing, decay, translation, and thus gene expression. ALKBH5 plays a crucial role in various human malignancies, mainly through m6A-dependent post-transcriptional regulation of oncogenes of tumor suppressors.

As an oncogene, ALKBH5 is upregulated in a variety of cancer tissues compared to non-cancerous tissues, including breast cancer (BC) (145), glioma and glioblastoma (146, 147), lung cancer (148, 149), ovarian cancer (150), acute myeloid leukemia (AML) (151, 152), GC (153), and colon cancer (154). In BC cells, the knockdown of ALKBH5 suppressed cell motility, cell colony formation, and migration ability (145). Breast cancer stem cells (BCSCs) are a small subpopulation of cells found in BC that are distinct from BC cells and can self-renew and differentiate into cancer cells. BCSCs resist chemotherapeutic agents and thus survive chemotherapy, resulting in tumor recurrence and metastasis (155). In BC, ALKBH5 can regulate the expression of NANOG, a core pluripotency factor. Overexpression of ALKBH5 reduces the m6A methylation level of NANOG mRNA in the 3’ untranslated region (3’UTR), resulting in increased stability and expression level of its mRNA. It also induced the enrichment of BCSCs in BC under hypoxic conditions (156). Another core pluripotency factor, KLF4, is positively regulated by ALKBH5-mediated m6A methylation in BC under the same mechanism (157). In glioma, a central nervous system malignancy, overexpression of ALKBH5 increases the transcriptional region m6A demethylation and thus enhances the mRNA stability of glucose-6-phosphate dehydrogenase (G6PD), the rate-limiting enzyme of the pentose phosphate pathway (PPP). Its increased expression promotes the oxidative flux of PPP, stimulating glioma cell growth (146). Glioblastoma stem cell-like cells (GSCs) are also present in glioblastoma and can maintain tumor growth and induce tumor recurrence after treatment (158). It has been shown that ALKBH5 demethylates nascent FOXM1 transcripts, which in turn promotes the interaction of the pre-mRNA of FOXM1 with the nuclear RNA binding protein HuR, leading to an increased expression and promoting the growth and self-renewal of GSCs (147). ALKBH5 enhances the radiation resistance of GSCs by regulating genes involved in homologous recombination and boosts the invasive ability of glioblastoma cells by upregulating the expression of YAP1 (159). Temozolomide is a first-line alkylating chemotherapeutic agent administered orally after surgical resection of glioblastoma, and resistance to it often occurs in the mid to late-stage of chemotherapy (160). There is evidence that ALKBH5 can enhance SOX2 expression by demethylating the mRNA of SOX2, thereby promoting resistance to Temozolomide in glioblastoma (161). In NSCLC, ALKBH5 is abnormally upregulated and closely associated with lower survival of patients. Mechanistically, ALKBH5 further promotes NSCLC cell proliferation and reduces apoptosis by inhibiting the stability of TIMP3 mRNA to reduce its translation level (148). In addition, UBE2C is an oncogene that inhibits autophagy and promotes the proliferation of NSCLC cells. Due to the upregulation of ALKBH5 in NSCLC, the number of m6A within UBE2C mRNA was reduced, resulting in its epi-transcriptional stabilization (162). Meanwhile, in KRAS mutant NSCLC, ALKBH5-induced m6A demethylation stabilized oncogenic drivers, including SOX2, SMAD7, and MYC, through the m6A reading protein YTHDF2 (163). However, another study showed that ALKBH5 could inhibit tumor cell proliferation and metastasis by directly reducing YTHDFs-dependent YAP1 expression in NSCLC and decreasing YAP1 activity through HuR-dependent control of miR-107/LATS2 expression (164). Moreover, ALKBH5 was upregulated in lung adenocarcinoma cells subjected to intermittent hypoxia. The abundance of m6A in FOXM1 transcripts was increased after the knockdown of ALKBH5 in lung adenocarcinoma cells, resulting in a reduction of FOXM1 translation and diminished cell proliferation and invasion (149). The ALKBH5-dependent m6A-demethylated lncRNA RMRP is also oncogenic in lung adenocarcinoma. Inhibition of RMRP in lung adenocarcinoma cell lines could achieve the same effects of ALKBH5 inhibition, including suppression of the abilities of cell proliferation, migration, and invasion, as well as promotion of apoptosis (165). In ovarian carcinoma, ALKBH5 expression was increased compared to non-cancerous ovarian tissues. Upregulation of ALKBH5 suppresses autophagy and promotes ovarian cancer cell malignancy by stabilizing BCL2 mRNA and promoting the binding of BCL2 to BECN1 (150). ALKBH5 could likewise increase the expression of NANOG mRNA by mediating its demethylation in ovarian cancer cells and enhancing its invasiveness through the NF-κB pathway (166). ALKBH5 is also significantly upregulated in endometrial carcinoma. ALKBH5 enhances the mRNA stability of IGF1R by demethylating its transcripts in the same way, promoting the translation of IGF1R, activating the IGF1R signaling pathway, and increasing the proliferation and invasive ability of endometrial cancer cells (167). In AML, ALKBH5 is upregulated, and its increased amount negatively correlates with patient survival. ALKBH5 enhances the malignancy of AML through post-transcriptional regulation of TACC3 and AXL (151, 152). However, TCGA data suggest that ALKBH5 is minimally expressed in AML, especially in individuals with TP53 mutations (151). In GC, overexpression of ALKBH5 decreased the m6A level of lncRNA NEAT1, which upregulated NEAT1 expression. upregulation of NEAT1 expression positively regulates the expression of EZH2, a polycomb repressive complex that acts as a scaffold to promote GC cell invasion and metastasis (153). Consistent with the findings in GC cells, the knockdown of ALKBH5 partially downregulated ALKBH5-associated NEAT1, inhibiting the proliferation and migration ability of CRC cells (154). In addition to these cancer types, ALKBH5 was found to be upregulated in RCC (168), HCC (169), osteosarcoma (170), esophageal epithelial cell carcinoma (171), cervical cancer (172) and oral epithelial cell carcinoma (173), suggesting that ALKBH5 is an oncogene in these cancers and that the amount of ALKBH5 upregulation is negatively associated with prognosis.

However, in other studies, ALKBH5 was shown to be down-regulated as a tumor suppressor, such as in bladder cancer and PC. In bladder cancer, downregulation of ALKBH5 was positively correlated with patient survival. Silencing ALKBH5 enhances the proliferation, migration, and invasion of bladder cancer cells and reduces their chemosensitivity to cisplatin *via* the CK2α-associated glycolytic pathway (174). In addition, ALKBH5 inhibited ITGA6 protein synthesis mediated by YTHDF1 and YTHDF3 by reducing m6A methylation in the 3’UTR of the ITGA6 transcript. Inhibition of ITGA6 expression further suppressed cell adhesion in bladder cancer (175). In PC, survival is improved in cases with high expression of ALKBH5 (176, 177). ALKBH5 controls KCNK15-AS1-dependent cell proliferation, migration, and invasive ability of PC through the demethylation of lncRNA KCNK15-AS1 (178). ALKBH5 also enhances post-transcriptional PER1 expression in an m6A-YTHDF2-dependent manner, activating the ATM-CHK2-P53/CDC25C signaling pathway and inhibiting PC cell proliferation (179). Moreover, overexpression of ALKBH5 exerts tumor suppressive effects in pancreatic ductal adenocarcinoma (PDAC) by reducing WIF1 mRNA methylation and regulating the Wnt pathway, leading to downregulation of C-MYC, Cyclin D1, MMP-2, and MMP-9 and sensitizing PDAC cells to gemcitabine (180). In clear cell renal cell carcinoma (ccRCC), ALKBH5 expression was positively correlated with the length of overall survival, indicating that ALKBH5 could be used as a prognostic biomarker (181). In HCC, ALKBH5 suppressed the ability of cancer cells to proliferate and invade through m6A-mediated inhibition of LYPD1 (182). Interestingly, ALKBH5 expression was significantly reduced in metastatic NSCLC. overexpression of ALKBH5 inhibited TGF-β-induced EMT and NSCLC cell invasion. Mechanistically, ALKBH5 affected the expression and mRNA stability of TGFβR2, SMAD3 and SMAD6 by erasing m6A modifications in NSCLC cells. This suggests that ALKBH5 can affect TGF-β-induced EMT in NSCLCs through the control of TGFβR2/SMAD3/SMAD6 signaling pathway (183).

### 4.6 ALKBH6

Since the crystal structure of ALKBH6 has only been resolved recently, its substrates have yet to be determined. The structure-based interaction partner screening not only identified transcriptional repressors ZMYND11 and ALKBH6 as having unknown interactions in suppressing tumor growth but also explained that histone modifications and nucleic acid modifications have cross-talk in epigenetic regulation (44). There is insufficient evidence for the substrates it can bind; however, its specific structure indicates the demethylase activity. In human PC cells, deletion of ALKBH6 leads to the accumulation of alkylating agents on DNA damage and significantly reduces cell survival. Furthermore, bioinformatic analysis of the TCGA database showed that overexpression of ALKBH6 provided better survival outcomes for pancreatic cancer patients. High expression of ALKBH6 was also detected in other types of adenocarcinomas, such as head and neck squamous cell carcinoma, RCC, and malignant melanoma (79). ALKBH6 is necessary to maintain genomic integrity and ensure cell survival (79).

### 4.7 ALKBH7

ALKBH7 is only found in mitochondria. Unlike other protective homologs of the AlkB family, ALKBH7 can accelerate programmed necrosis following exposure to alkylating or oxidizing agents by inhibiting the function of mitochondria. Bioinformatic analysis has shown that elevated ALKBH7 expression can be detected in most cancers compared to normal tissues, such as HCC (144), lung adenocarcinoma (184), and serous ovarian carcinoma (185). The upregulated ALKBH7 is also associated with the infiltration of immune cells, such as CD4+ T cells, CD8+ T cells, macrophages, and neutrophils. A pan-cancer analysis showed that ALKBH7 expression correlated with the expression of immune checkpoint (ICP) genes in many cancers and was mainly negatively correlated (186). The ALKBH7 gene co-expression network regulated cellular immune, oxidative, phosphorylation, and metabolic pathways (186). However, these studies are all focused on bioinformatic analysis. In-depth research on the ALKBH7 signal pathway network still needs to be completed.

### 4.8 ALKBH8

ALKBH8 has unique RRM and MTase domains that prefer to bind to some specific tRNAs, such as tRNA (Arg), tRNA (Gly), and tRNA (Glu), but not tRNA (Lys), to function as a methyltransferase and thus regulate the protein synthesis process (86, 87). There are few studies on the role of ALKBH8 in the signaling pathway of cancer cells. In urothelial cancer cells, silencing ALKBH8 leads to the downregulation of NAD(P)H oxidase-1 (NOX-1), which reduces the production of reactive oxygen species (ROS) and induces apoptosis through subsequent activation of c-jun NH2-terminal kinase (JNK) and p38. Activation of JNK and p38 also leads to the phosphorylation of H2AX (γH2AX), a variant of mammalian histone H2A (93). Silencing of ALKBH8 also significantly inhibited the invasion, angiogenesis, and growth of bladder cancer cells by downregulating the protein expression of survivin, an anti-apoptotic factor, which is upregulated in bladder cancer (187).

### 4.9 FTO

As an obesity-associated protein, early research on the relationship between FTO and cancer mainly emphasized obesity-associated gene polymorphisms (188), and there was no detailed study of signaling pathways. Therefore, the types of cancer studies are also focused on obesity-related cancers, such as BC (189), PC (190), and CRC (191). Only after recognizing FTO and ALKBH5 as mRNA demethylases have their epigenetic regulatory roles in cancers been gradually emphasized (12, 192). As a result, there are now many studies on the involvement of FTO in regulating cellular pathways in different types of cancer.

FTO and ALKBH5 are RNA demethylases, but their biological functions differ (12). While ALKBH5 only catalyzes the removal of m6A from ssRNAs (74), FTO has multiple substrates. For example, FTO can demethylate m6A on the RRACH motifs of mRNAs and long non-coding RNAs (lncRNAs) (193), as well as N6,2 O-dimethyl adenosine (m6Am) on mRNA and snRNAs, m1A on tRNAs, 3-methyl thymidine (m3T) on ssDNA, and 3-methyl uracil (m3U) on ssRNA (12, 94, 96, 107). However, in tumor regulation, RNA m6A remains the primary substrate for FTO in cells, as there are many more m6A sites in RNA transcripts than m6Am sites (194, 195). m6A modification in mRNA by FTO affects mRNA processing, stability, and translation, and dysregulation of FTO is seen in many types of cancer.

FTO is highly expressed in many cancers, and its high expression is positively associated with shorter overall survival. In specific AML, FTO is aberrantly overexpressed and thus exerts oncogenic effects. For example, FTO can inhibit myeloid differentiation and induce leukemogenesis by demethylating the mRNA m6A of SOCS box protein 2 (ASB2) and retinoic acid receptor alpha (RARA), which leads to degradation of their mRNAs and triggering the corresponding signaling pathways. Also, FTO can reduce AML cells’ sensitivity to all-trans retinoic acid by inhibiting the expression of ASB2 and RARA (196). R-2-hydroxyglutarate (R-2HG) is a highly abundant oncometabolite produced by mutant isocitrate dehydrogenase 1/2 (IDH1/2). R-2HG inhibits FTO protein activity, resulting in elevated global mRNA m6A levels in R-2HG-sensitive leukemia cells. In turn, high mRNA m6A levels reduce the stability of MYC/CEBPA transcripts, leading to the inhibition of related signaling pathways. R-2HG has broad anti-leukemic activity *in vivo* and *in vitro*, inhibiting the proliferation and viability of leukemic cells and promoting cell cycle arrest and apoptosis. High expression of FTO sensitizes leukemic cells to R-2HG; in contrast, over-activation of MYC signaling enhances resistance to R-2HG. Similarly, R-2HG exhibited antitumor activity in gliomas (195). FTO is overexpressed in leukemia stem cells (LSCs), allowing the stable expression of many oncogenes. Knockdown of FTO or inhibition using small molecule inhibitors can significantly impair the self-renewal ability of LSCs and reprogram the immune response by suppressing the expression of ICP genes, especially LILRB4 (197). In BC, FTO is in an up-regulated state. Upregulated FTO mediates m6A demethylation in the 3’UTR of BNIP3 mRNA in the FoxO signaling pathway and induces its degradation through a mechanism independent of YTHDF2, thereby promoting BC cell proliferation, cell colony formation, and metastasis (198). Furthermore, FTO upregulated ADP-ribosylation factors, such as GTPase 5B (ARL5B), by inhibiting miR-181b-3p, thereby increasing the invasive and migratory capacity of BC cells (199). In NSCLC, FTO facilitates cancer cell proliferation by activating KRAS signaling or the upregulation of MZF1 or USP7 in an m6A-dependent way (200–202). In HCC, the downregulation of FTO can suppress the expression of SOX2, NANOG, and KLF4 transcripts, reducing the pluripotency of HCC cells. And the activity of FTO can also be regulated by AMD1 (203). In PC, FTO is upregulated compared to normal human pancreatic ductal epithelial cells. Downregulation or inhibition of FTO leads to diminished PC cell proliferation, invasion, and EMT through cell cycle arrest in the G (1) phase and induction of p21cip1 and p27kip1. This is associated with reduced tumor stem cell gene expression and impaired tumorsphere formation (204). In GC, FTO also promotes GC by stabilizing MYC by eradicating m6A in the 5′UTR of MYC (205). In CRC, FTO enhances MYC expression by removing m6A modifications, leading to enhanced proliferation and invasion of CRC cells (206). In cervical squamous cell carcinoma (CSCC), FTO upregulates the translation of two typical oncogenic transcripts, E2F1 and MYC, in an m6A-dependent manner, thereby promoting cell proliferation and migration (207). In endometrial cancer, FTO catalyzes the demethylation of the 3’UTR region of HOXB13 mRNA, which reduces the decay of mRNA associated with the recognition of m6A modification of YTHDF2 protein and increases the expression of HOXB13 protein, accompanied by the activation of the WNT signaling pathway and the expression of downstream proteins, leading to tumor metastasis and invasion (208). In ovarian cancer, FTO was upregulated. Overexpression of FTO increased the survival and autophagy of ovarian cancer cells but decreased apoptosis. Overexpression of FTO also promoted the phosphorylation of AKT (209). Furthermore, by reducing the m6A level of 3’UTR and mRNA stability of two phosphodiesterase genes (PDE1C and PDE4B), FTO enhanced second messenger cAMP signaling and inhibited the stemness characteristics of ovarian cancer cells (210). In bladder cancer, FTO accelerates tumorigenesis by removing m6A modifications and activating the MALAT1/miR-384/MAL2 cascade in MALAT1 mRNA (211).

Like ALKBH5, FTO is downregulated as a tumor suppressor in a variety of cancers, including BC (145), lung cancer (212), CRC (213), bladder cancer (214, 215), prostate cancer (216), ccRCC (181, 217, 218), and adrenocortical cancer (219). In BC, FTO depletion triggers the EMT program by upregulating mRNA m6A levels and altering the 3’ end processing of key mRNAs in the Wnt signaling cascade (220). In lung cancer, Wnt signaling induces EZH2 to form a protein complex with β-catenin and bind the LEF/TCF binding element in the FTO promoter region. The presence of EZH2 also upregulates H3K27me3 and thereby suppresses FTO expression. Downregulation of FTO significantly increases the m6A levels of many genes in critical pathways, especially metabolic pathway genes, such as MYC. Elevated m6A levels on MYC mRNA promote the binding of YTHDF1, thereby promoting its translation and increasing glycolysis, tumor cell proliferation, and tumorigenesis (212). In CRC, methylated metastasis-associated protein 1 (MTA1) mRNA can be recognized by an m6A “reader,” insulin-like growth factor 2 mRNA-binding protein 2 (IGF2BP2), and become more stable. FTO, in turn, can exert tumor suppressive effects by destabilizing MTA1 by removing its m6A (213). In intrahepatic cholangiocarcinoma, FTO disrupts the transcripts of the oncogene TEAD2 in the same way as suppressing tumor progression (221). Interestingly, FTO-mediated demethylation of different regions of bladder cancer mRNA exerted differential expression of various genes. Demethylation of the 3′ UTRs of CSNK2A2 and ITGA6 transcripts stabilized these mRNAs. In contrast, demethylation of the 5′ UTRs of MALAT1 and NOTCH1 transcripts reduced the expression of their transcripts, thereby generally impairing the proliferation and invasive capacity of tumor cells (214).

## 5 Summary and prospective

There are nine homologs of the AlkB family, ALKBH1-8 and FTO, distributed within different cell locations. The crystal structure of ALKBH4 still needs to be resolved. As more and more AlkB homologs and substrates crystal structures are being determined, their biochemical functions are gradually understood. The nine homologs can catalyze the demethylation of different substrates, such as DNA, RNA, and histone, affecting DNA repair, replication, and gene expression. Aberrant gene expression correlated with cancer cell proliferation, migration, and invasion. The roles of AlkB homologs in cancers through epigenetic regulation have received increasing attention from scholars.

### 5.1 ALKBH5 and FTO

Although ALKBH5 and FTO are in different branches of the phylogenetic tree (see **Figure 2**), both function as RNA demethylase. By removing m6A from mRNA, ALKBH5 and FTO can affect pre-mRNA processing, mRNA stability, and translation and regulate numerous pathways.

Both ALKBH5 and FTO can regulate the cancer epigenome through demethylation of mRNA m6A, leading to changes in the ability of cells to survive, proliferate, invade, and metastasize as alterations in drug sensitivity, cancer stem cell status, and cancer immunity. Although much research has been done on ALKBH5 and FTO, however, there are still some questions that need to be addressed. Firstly, it is worth noting that ALKBH5 and FTO act as oncogenes or tumor suppressors in some cancers. These contradictory roles in specific cancer types may be due to limited sample sizes, different assays, high heterogeneity of cancer samples (cell lines and primary tumor samples), and other potential biases. Therefore, an improved methodology is needed to fully explore the exact context of the role of ALKBH5 and FTO in cancer, especially for those cancers where the role of ALKBH5 and FTO is highly controversial. Secondly, it is plausible that m6A methyltransferase and demethylase exhibit opposing roles in specific pathologies.

Nevertheless, there are exceptions. For example, writers and erasers of m6A, including METTL3, METTL14, ALKBH5, and FTO, are all abnormally upregulated in AML and have primary oncogenic functions (151, 152, 196, 222, 223). Further investigations are needed to clarify why enzymes that catalyze opposite reactions can have similar roles in specific cancers.

Targeting dysregulated ALKBH5 and FTO is an appealing therapeutic approach for cancer in light of their essential functions in various malignancies. Several biopharmaceutical companies have successfully developed small molecule inhibitors of FTO with high efficacy and selectivity, and some of them have achieved promising preliminary results in preclinical investigations (101, 195, 224, 225). However, targeting ALKBH5 as an m6A eraser for clinical applications is much less studied than on FTO. Although several ALKBH5 inhibitors have been identified, the issues of potency, selectivity, and cytotoxicity are still urgent to address. Targeting tools developed using CRISPR towards m6A can achieve m6A-targeted regulation against oncogenes or tumor suppressors. The ⍺KG binding pockets of ALKBH5 and FTO are distinct, facilitating the development of selective compounds targeting these two RNA demethylases. Crystallographic and biochemical profiling using various ⍺KG analogs indicated that the active site cavity of ALKBH5 is significantly smaller than FTO, so ALKBH5 preferentially binds small molecule inhibitors (43, 75).

### 5.2 ALKBH1, ALKBH2 and ALKBH3

ALKBH1 has multiple substrates, including ssDNA, tRNA, dsDNA, and mRNA, among which N6mA on genomic DNA is most important. The N6mA methylation of genomic DNA co-localized with heterochromatin histone modification, altering the chromatin accessibility and further regulating gene transcription (52). Several studies on the roles of ALKBH1 in cancer as a genomic DNA N6mA demethylase, such as glioblastoma, TSCC, HCC, and GC. However, the parts played are different. It may be related to tumor heterogeneity and other downstream genes regulated. In addition, as mRNA demethylase, it can directly control the translation of genes (119), even the methyltransferase gene (120). In the future, more efforts could be put into the role of DNA N6mA demethylase. By the more advanced approaches like bioinformatics, and single-cell sequencing, we can find the N6mA gene loci bound by ALKBH1 in different cancers and the pathways regulated.

ALKBH2 and ALKBH3 exist in the same clade of the phylogenetic tree. ALKBH2 prefers dsDNA, while ALKBH3 prefers ssDNA and mRNA. As a dsDNA demethylase, the role of ALKBH2 in cancers is only focused on phenotype, and the pathways by which downstream genes are up or down-regulated are unclear. ALKBH3 is identical to PCA-1 (126, 127), and its activity can be measured by MAQ probes (128). Thus, it is relatively more studied in cancer.

ALKBH3 inhibitors also effectively inhibit the development and invasive ability of cancer cells implanted *in vivo* (130, 131). However, like ALKBH2, as ss DNA demethylase, there is no direct evidence of how ALKBH3 affects gene expression by regulating ssDNA demethylation. Instead, as mRNA m1A demethylase, ALKBH3 has been identified in specific cellular pathways due to its ability to affect gene expression directly. Also, ALKBH3 is a tRNA m1A, m3C, and m6A demethylases. The demethylation of tRNA increases the rate of protein synthesis.

So far, the roles of ALKBH1, ALKBH2, and ALKBH3 in cancer regulation have not been studied much. It is unclear which part dominates in different tumors for ALKBH1 and ALKBH3, which have both DNA and RNA demethylase functions. More studies are needed to reveal their panoramic view of cancer.

### 5.3 ALKBH4, ALKBH6, ALKBH7 and ALKBH8

The crystal structure of ALKBH4 has yet to be resolved, and its substrate is currently found only as actin lys-84 (K84me1). ALKBH4, by its presumed structural characteristics, prefers to bind and catalyze proteins. There is evidence that ALKBH4 can competitively bind to WDR5, a vital component of the histone methyltransferase complex, to regulate H4K4me3 histone methylation (143). Meanwhile, ALKBH4 can act synergistically with ALKBH1 to increase the genomic N6mA (226). It is reasonable that ALKBH4, as a histone demethylase, could perform synergistically with genomic DNA demethylase to regulate gene accessibility and thus affect gene translation.

Although the crystal structure of ALKBH6 has been resolved, its substrate still needs to be clarified. According to its structural features, ALKBH6 should have a demethylation effect, but the substrate is yet to be found. Nevertheless, there is evidence that ALKBH6 plays an essential role in maintaining genomic integrity, particularly in cancer (79).

ALKBH7 is the only member of the AlkB family in mitochondria, and its function may be related to regulating energy metabolism and apoptosis-related diseases (80). Based on its protein structure, the substrates of ALKBH7 are presumed to be proteins. Furthermore, a pan-cancer analysis showed that ALKBH7 expression correlates negatively with the expression of ICP genes in many types of cancer and that ALKBH7 gene co-expression networks regulate cellular immune, oxidative, phosphorylation, and metabolic pathways (186). However, these studies have focused on bioinformatic analysis. Therefore, studying its substrates can clarify the role played by ALKBH7 in mitochondria and thus provide a deeper understanding of its regulatory role in cancer.

The primary substrate of ALKBH8 is some specific tRNAs. Its structure suggests that ALKBH8 has both an additive dioxygenase role and a structural domain of C-terminal methyltransferase (MTase), a form that complexes with the accessory protein Trim112 to methylate cm5U and produces mcm5U at the wobbly position tRNA, which is the final and most crucial post-transcriptional modification of the anticodon loop in the target tRNA (85, 88, 89). ALKBH8 has only been studied in bladder cancer. Like ALKBH1, ALKBH3, as enzymes that can bind to tRNA and affect cancer prognosis by regulating protein synthesis, is also a new research direction.

## 6 Conclusion

Overall, we summarized the research progress and therapeutic potential of AlkB homologs in cancer. As an emerging family of epigenetic regulatory enzymes, their structures, biochemical responses, and cellular signaling pathways remain unclarified. AlkB homologs may offer new hope for diagnosing and treating tumors after more in-depth studies.

## Author contributions

QL completed the writing and drawing. QL and QZ developed the overall thinking of the paper. QZ revised the paper. All authors contributed to the article and approved the submitted version.
